# TM3 and STM3 Promote Flowering Together with FUL2 and MBP20, but Act Antagonistically in Inflorescence Branching in Tomato

**DOI:** 10.3390/plants12152754

**Published:** 2023-07-25

**Authors:** Iris E. Zahn, Chris Roelofsen, Gerco C. Angenent, Marian Bemer

**Affiliations:** 1 Laboratory of Molecular Biology, Wageningen University & Research, 6708 PB Wageningen, The Netherlands; iris.zahn@wur.nl (I.E.Z.); gerco.angenent@wur.nl (G.C.A.); 2 Business Unit Bioscience, Wageningen University & Research, 6708 PB Wageningen, The Netherlands

**Keywords:** *Solanum lycopersicum*, floral transition, inflorescence architecture, reproductive meristems, MADS-box transcription factor, SUPPRESSOR OF OVEREXPRESSION OF CONSTANS 1, FRUITFULL

## Abstract

The moment at which a plant transitions to reproductive development is paramount to its life cycle and is strictly controlled by many genes. The transcription factor SUPPRESSOR OF OVEREXPRESSION OF CONSTANS 1 (SOC1) plays a central role in this process in *Arabidopsis*. However, the role of SOC1 in tomato (*Solanum lycopersicum*) has been sparsely studied. Here, we investigated the function of four tomato *SOC1* homologs in the floral transition and inflorescence development. We thoroughly characterized the *SOC1*-like clade throughout the Solanaceae and selected four tomato homologs that are dynamically expressed upon the floral transition. We show that of these homologs, TOMATO MADS 3 (TM3) and SISTER OF TM3 (STM3) promote the primary and sympodial transition to flowering, while MADS-BOX PROTEIN 23 (MBP23) and MBP18 hardly contribute to flowering initiation in the indeterminate cultivar Moneyberg. Protein–protein interaction assays and whole-transcriptome analysis during reproductive meristem development revealed that TM3 and STM3 interact and share many targets with FRUITFULL (FUL) homologs, including cytokinin regulators. Furthermore, we observed that mutating *TM3/STM3* affects inflorescence development, but counteracts the inflorescence-branching phenotype of *ful2 mbp20*. Collectively, this indicates that TM3/STM3 promote the floral transition together with FUL2/MBP20, while these transcription factors have opposite functions in inflorescence development.

## 1. Introduction

The developmental switch to flowering requires an identity change in the shoot apical meristem (SAM) [1]. In tomato (*Solanum lycopersicum*), this switch underlies important agricultural traits such as flowering time and inflorescence architecture. When progressing from the vegetative to the reproductive stage, the flat and vegetative tomato SAM forms a dome-like structure and gradually changes into a transition meristem (TM) and subsequently floral meristem (FM). The FM terminates the shoot by producing a flower, and gives rise to a new inflorescence meristem (IM) on its flank. The IM will develop into an FM and form a new IM, and so forth. This iterative process results in a zigzagged inflorescence. Inflorescence branching depends on the development of the different reproductive meristems (TM, FM, IM). When FM maturation is delayed, multiple IMs are formed on the flank of a single FM, resulting in a (sometimes highly) branched inflorescence [2,3]. Thus, the development of the apical meristem determines both flowering time and inflorescence architecture.

A tomato shoot repeatedly makes the transition to flowering due to its sympodial architecture. After the primary apex terminates in an inflorescence, a new vegetative shoot is formed from the sympodial meristem (SYM), derived from the axillary meristem of the youngest leaf. The vegetative SYM again has to transition to flowering, thereby terminating the sympodial shoot. This sequential process is repeated infinitely in indeterminate cultivars or finitely in determinate cultivars [2,3].

A complex gene regulatory network underlies the development of the reproductive meristems. To induce the floral transition, *SINGLE FLOWER TRUSS* (*SFT*, homolog of *FLOWERING LOCUS T* (*FT*)) and *FALSIFLORA* (*FA*, homolog of *LEAFY* (*LFY*)) are essential. *SFT* encodes the florigen, a systemic signal that moves from the leaves to the SAM to induce flowering in primary and sympodial shoots [4,5]. In a parallel pathway, *FA* also induces flowering by regulating FM identity. Consequently, flowering is abolished when disrupting both *SFT* and *FA* [4,6]. Other promoters of the floral transition, albeit having a mild role compared to *SFT* and *FA*, are the *SUPPRESSOR OF OVEREXPRESSION OF CONSTANS 1* (*SOC1*) homologs *TOMATO MADS 3* (*TM3*) and *SISTER OF TM3* (*STM3*) [7], and the *FRUITFULL* (*FUL*) homologs *FUL2* and *MADS-BOX PROTEIN 20* (*MBP20*) [8].

After the initiation of flowering, a switch to IM and FM identity is required for inflorescence development. To confer reproductive fate, IM identity is promoted by *MACROCALYX* (*MC*, homolog of *APETALA 1* (*AP1*)) [9]. Concomitantly, several genes promote the development of IMs into FMs, thereby repressing inflorescence branching. These genes include *COMPOUND INFLORESCENCE* (*S*, homolog of *WUSCHEL-RELATED HOMEOBOX 9* (*WOX9*)) and *ANANTHA* (*AN*, homolog of *UNUSUAL FLORAL ORGANS* (*UFO*)), and the *SEPALLATA 4* (*SEP4*) homologs *JOINTLESS 2* (*J2*) and *ENHANCER OF J2* (*EJ2*), of which the mutants have highly branched inflorescences [3,10,11]. Similar to *j2 ej2*, mutations in the floral regulators *FUL1*, *FUL2* and *MBP20* result in enhanced inflorescence branching [8].

Given that *SOC1* is a master regulator of flowering time in *Arabidopsis* [12] and has conserved functions in a wide range of other species [13,14,15,16,17,18,19], there is still little known about its role in tomato. Two tomato *SOC1* homologs, *TM3* and *STM3*, are located in a highly repetitive and complex genomic region [7]. In addition, no research has yet been performed to elucidate the function of putative other tomato *SOC1*-like genes, and a complete phylogenetic analysis to identify and characterize all tomato *SOC1*-like genes is still lacking. 

MADS-domain transcription factors (TFs) are involved in numerous developmental processes and often function as heterodimeric or -tetrameric complexes [20,21]. Recent publications have unveiled functions of individual MADS proteins in tomato flowering, including *SOC1*-, *FUL*- and *SEP*-like genes [7,8,9,10,11,22,23]. However, it is still largely unclear in which complexes these proteins interact to regulate the different steps during the floral transition and inflorescence development. In *Arabidopsis*, SOC1 and FUL form a strong heterodimer. The proteins interact in yeast [24] and they bind to the same promoter elements [25], preferentially as a heterodimer or -tetramer and not as homodimers [26]. Moreover, genetic experiments show that *SOC1* and *FUL* function partially redundantly in flowering time [27]. Together, this indicates that SOC1 and FUL physically interact in *Arabidopsis* to control several developmental processes, including the floral transition. 

It is likely that tomato SOC1 homologs genetically and physically interact with the aforementioned FUL and SEP4 homologs to regulate target gene expression in the reproductive meristems. Previous publications showed that the highly branching inflorescence phenotype of the *j2 ej2* mutant can be largely suppressed by a knock-out of *TM3* and *STM3* [7,22,28], and that *STM3* and *J2* compete for binding at the *FUL1* promoter [28]. These findings indicate that tomato *SOC1* homologs can counteract the function of *J2* and *EJ2*. Furthermore, FUL2 can interact with TM3, STM3, J2 and EJ2 [8]. STM3 can also interact with J2 [28], supporting that these TFs form heteromeric complexes to fulfill their functions. Strikingly, it was also reported that STM3 activates FUL1 in the apex [22,28] and that a knock-out of *FUL1* can, like *TM3* and *STM3*, repress the highly branching phenotype of *j2 ej2* [22]. This apparently contradicts the repressive function in inflorescence branching of *FUL* homologs in a WT background [8], showing that the interactions of MADS-domain proteins during reproductive meristem development are still far from understood. Thus, it is clear that SOC1, FUL, and SEP4 homologs play a role in the regulation of the floral transition and inflorescence development in tomato, but their independent and combinatorial functions are still unclear. Here, we shed more light on the genetic and physical interactions of *SOC1* and *FUL* homologs, and the functions of their encoded proteins during the floral transition and inflorescence development.

We characterized the developmental roles of the meristem-expressed *SOC1* homologs *TM3*, *STM3*, *MBP23* and *MBP18* during reproductive meristem development in the indeterminate cultivar Moneyberg. Using CRISPR/Cas9 mutagenesis and whole-transcriptome profiling, we show that of these four homologs, mainly *TM3* and *STM3* are important regulators of the floral transition and inflorescence development, while *MBP18* appears to play a minor role. We found that TM3/STM3 most likely control the floral transition by interacting with FUL2/MBP20 in a protein complex and together repress cytokinin inhibitors. Subsequently, TM3/STM3 and FUL1/FUL2/MBP20 have antagonistic functions in specifying FM and IM identity, which is required for normal inflorescence development.

## 2. Results

### 2.1. Identification and Characterization of Tomato SOC1 Homologs

#### 2.1.1. Phylogenetic Analysis of the Solanaceae SOC1-like Proteins

**Figure 1:**
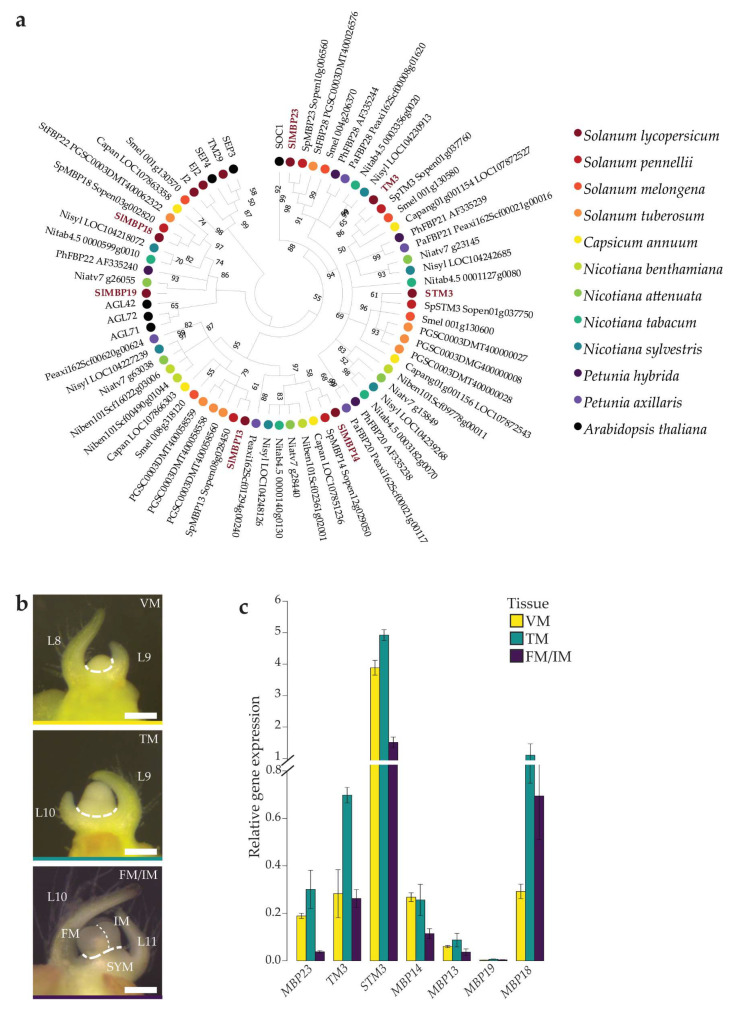
Characterization of tomato *SOC1*-like genes during the floral transition. (**a**) Phylogenetic tree of SOC1-like proteins from several Solanaceae species and *Arabidopsis thaliana*. Numbers at the branches indicate bootstrap values inferred from 5000 replicates. (**b**) Stereomicroscope images of reproductive meristem development. Dashed lines indicate hand-dissected tissue for transcriptome analysis. The scale bar is 200 µm. (**c**) Expression of the *SOC1*-like genes *MBP23*, *TM3*, *STM3*, *MBP14*, *MBP13*, *MBP19* and *MBP18* relative to *CAC* in WT VM, TM and FM/IM tissue (dCT values are shown per gene relative to the reference). Shown are the mean values ± SE of three biological replicates. L, leaf; VM, vegetative meristem; TM, transition meristem; FM/IM, floral and inflorescence meristem; SYM, sympodial shoot meristem.

To characterize the complete *SOC1*-like clade in tomato, a phylogenetic tree was built based on related proteins in several Solanaceae species and *Arabidopsis* (Figure 1a). The tree shows two clades of homologous proteins that are evolutionary most closely related to AtSOC1. Most analyzed Solanaceae have a single homolog in the first clade, including the FBP28-like proteins from *Petunia*. The tomato protein in this clade is encoded by *Solyc10g017640* (coding for the MADS domain) and *Solyc10g017630* (coding for the rest of the protein), of which the latter locus has been named MBP23 [29]. Homologs from *Capsicum annuum*, *Nicotiana benthamiana* and *N. attenuata* are absent in this clade. The other clade closely related to AtSOC1 contains the tomato paralogs TM3 and STM3. Tomato, eggplant, pepper, *N. attenuata*, *N. tabacum* and *N. sylvestris* each have two paralogs in this clade, which are separated in TM3- and STM3-like clusters. Potato and *N. benthamiana* do not have TM3-like proteins, while the *Petunia* FBP20 proteins group outside of the TM3/STM3 clade. More distantly related to AtSOC1 is a clade with the tomato homologs MBP13 and MBP14, which form separate clusters. Tomato, pepper, most *Nicotiana* species and *Petunia* have at least one homolog in both clusters, while eggplant and potato only have MBP13-like homologs.

To identify all proteins that potentially function as SOC1-like TFs in the regulation of tomato flowering, homologs of the related *Arabidopsis* flowering genes *AGAMOUS-LIKE 42* (*AGL42*), *AGL71* and *AGL72*, which emerged from a duplication of the ancestral *SOC1* gene, were included in the phylogenetic analysis [30]. For most Solanaceae analyzed here, a single homolog is present in the clade containing AGL42, AGL71 and AGL72. Only for *S. lycopersicum*, two proteins, MBP18 and MBP19, group to this clade. Finally, floral regulators from the SEP family were used to root the tree.

It has recently become clear that structural variation, often involving gene copy number variation, is a common phenomenon within the germplasm of crops [7,31,32,33]. In tomato, *TM3* and *STM3* are located in a repetitive genomic region that is variable in *STM3* copy number across tomato cultivars [7]. In our study, we used the indeterminate variety Moneyberg (a TMV-resistant version of Moneymaker [34]). A recent genome assembly of the cultivar Moneymaker contains a single copy of *TM3* and two copies of *STM3* [35], indicating that Moneyberg also carries two *STM3* copies (Supplementary Figure S1a). The coding sequences of *TM3* and *STM3* are highly similar and their MADS domains are even identical at the nucleotide level (Supplementary Figure S1b). Consequently, we can distinguish *TM3* from *STM3*, but cannot discriminate between the two *STM3* copies on a DNA or RNA level.

#### 2.1.2. Expression of Tomato SOC1 Homologs Peaks during the Primary Floral Transition

To select the homologs that potentially fulfil the flowering function of *AtSOC1* in tomato, we characterized the expression patterns of the identified tomato *SOC1*-like genes during reproductive meristem development using qPCR on cDNA from three different meristem stages: VM, TM and combined FM/IM of the primary inflorescence (Figure 1b,c). We found a clear peak in expression in the TM for *MBP23*, *TM3*, *STM3* and *MBP18*. Of these genes, *STM3* stood out because of its extremely high expression levels. In contrast, expression was weak or decreasing during reproductive meristem development for *MBP14*, *MBP13* and *MBP19*. Therefore, based on phylogenetics combined with considerable and dynamic expression in the TM, we selected *MBP23*, *TM3*, *STM3* and *MBP18* as targets to further study the role of *SOC1*-like genes during reproductive meristem development in tomato.

### 2.2. Functional Characterization of Tomato SOC1 Homologs in Flowering

#### 2.2.1. TM3 and STM3 Display a Broad Protein–protein Interaction Profile, in Contrast to MBP23 and MBP18

**Figure 2:**
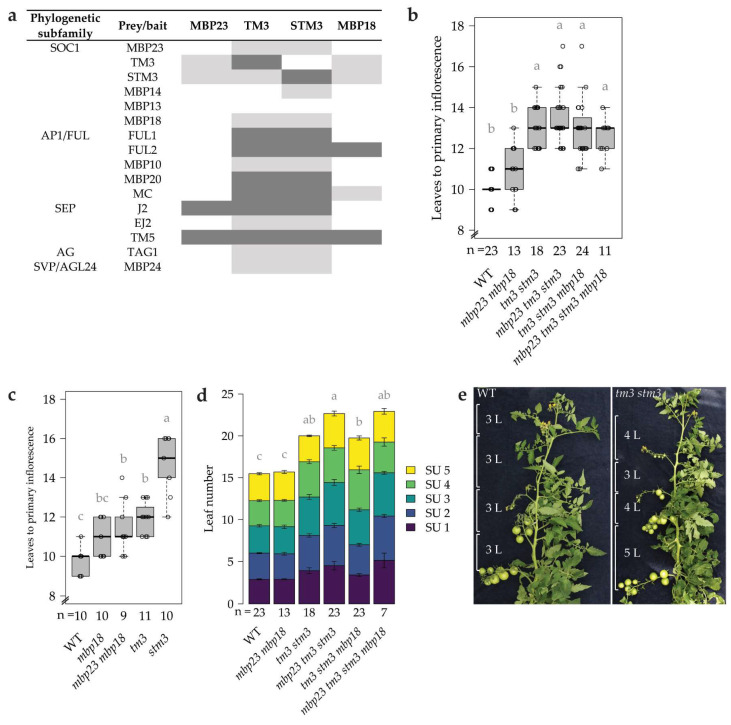
Functional characterization of SOC1 homologs in flowering control. (**a**) Y2H screening showing interactions of SOC1-like proteins with several MADS-domain TFs. Reciprocal interaction is shown by dark grey shading, one-way interaction by light grey shading, and blank cells indicate a failure to interact. MBP24 was only tested as prey (AD), not as bait (BD). (**b**) Quantification of the number of leaves to the first inflorescence, i.e., the primary transition to flowering, in higher-order *slsoc1* mutants under greenhouse conditions. (**c**) Quantification of the primary transition to flowering in single and double *slsoc1* mutants under growth-chamber conditions. (**d**) Quantification of the sequential sympodial shoot floral transitions in higher-order *slsoc1* mutants under greenhouse conditions. Shown are mean values of the cumulative number of leaves per sympodial unit (SU) ± SE. The average size of the first five SUs per plant was used to calculate significance. (**e**) Representative sympodial shoots were from WT and *tm3 stm3* plants. Leaves were bent to the right for visualization. L, leaf number. In (**b**–**d**), letters indicate statistical significance.

To gain more insight into the overlapping and specific functions of the four selected tomato SOC1 homologs, we investigated their interactions with several other tomato MADS-domain proteins linked to flowering [8,9,10,11,36,37]. Yeast two-hybrid (Y2H) assays showed a broad and largely overlapping protein–protein interaction profile for TM3 and STM3, which was clearly distinguishable from the interaction profiles of MBP23 and MBP18 (Figure 2a and Supplementary Figure S2). TM3 and STM3 interacted with all screened flowering regulators from the AP1/FUL, SEP, AGAMOUS (AG) and SHORT-VEGETATIVE PHASE (SVP)/AGL24 TF families. Only the varying ability to heterodimerize with other SOC1 homologs illustrates minor differences between TM3 and STM3. Although both TM3 and STM3 could homodimerize in yeast, interaction was much stronger for STM3. These extensive interaction profiles indicate a broad role for TM3 and STM3 in reproductive development. Furthermore, our assays showed that MBP23 and MBP18 have a more limited number of protein interaction partners. Their interactions are very similar, except that MBP18 interacted with the important flowering regulators FUL2 and MC, while MBP23 did not. Both MBP23 and MBP18 were unable to form homodimers; thus, these proteins most likely depend on interaction with other MADS-domain proteins to fulfil their functions.

#### 2.2.2. TM3 and STM3 Promote the Primary and Sympodial Transition to Flowering

To further characterize the functions of the four selected tomato SOC1-like TFs *in planta*, we generated single- and higher-order mutants in the cultivar Moneyberg by CRISPR/Cas9 mutagenesis. We targeted the MADS-box sequence using two sgRNAs per gene and sgRNAs for multiple genes were combined in transformations to generate higher-order mutants with independent mutations. Of the quadruple mutant *mbp23 tm3 stm3 mbp18*, an additional independent line was generated by crossing *mbp23 tm3 stm3* with *tm3 stm3 mbp18* and selecting homozygous F2 *mbp23 tm3 stm3 mbp18* plants. *TM3* and *STM3* were targeted simultaneously in higher-order mutants, as their MADS boxes are identical. The single mutants *tm3* and *stm3* were obtained by targeting their unique second exon. Primary transformants with deleterious mutations in all targeted genes were selected, and T1/T2 offspring with homozygous mutations in *MBP23*, *TM3* and *MBP18*, and homozygous or multiallelic mutations in the four alleles of *STM3* were further analyzed. The obtained genotypes are shown in Supplementary Figure S3.

Phenotyping of the selected mutants revealed that *TM3* and *STM3* promote the primary transition to flowering (Figure 2b and Supplementary Figure S4). Initially we disrupted these two genes simultaneously, which delayed flowering time by three leaves, confirming the observation of Alonge et al. [7] in the determinate cultivar M82. The newly characterized *SOC1*-like genes *MBP23* and *MBP18* hardly affected flowering time. We did not observe statistically significant effects on flowering time for the double mutant, nor additively when stacking mutations in the *tm3 stm3* mutant. Nevertheless, there was a trend towards mildly delayed flowering in *mbp23 mbp18*. To compare flowering times more precisely, we performed a second screening in a growth chamber with more stable and controlled conditions than in the greenhouse of the initial screening. We characterized the single mutants *tm3*, *stm3* and *mbp18*, and the double mutant *mbp23 mbp18* (Figure 2c). This revealed that *TM3*, and more strongly also *STM3*, promote flowering independently, and that *MBP18* mildly contributes to the primary floral transition, while we cannot exclude there is a mild role for *MBP23* as well.

Similar to the primary transition, the sympodial transition to flowering was delayed in *tm3 stm3* mutants (Figure 2d,e). While the number of leaves per sympodial unit (SU) is consistently three in the Moneyberg WT, the *tm3 stm3* mutants displayed a mild but significant increase in leaf number in the SUs. *MBP23* and *MBP18* did not affect sympodial leaf number, indicating that these genes have a minor role in the primary, but not in the sympodial transition to flowering.

### 2.3. SlSOC1 and SlFUL Genes Regulate a Set of Common Target Genes during the Floral Transition

**Figure 3:**
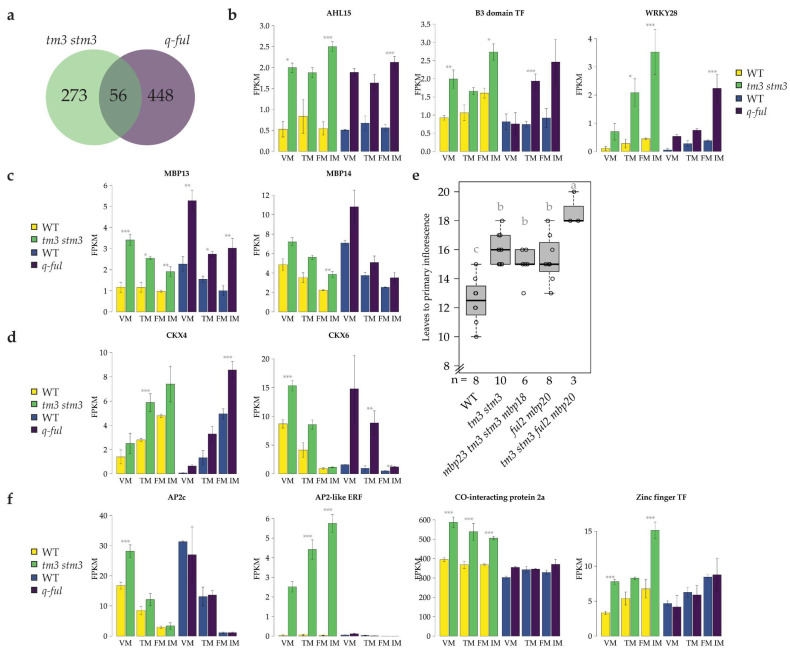
*TM3/STM3* share targets with the *SlFULs* that also independently contribute to the floral transition. (**a**) Venn diagram showing the overlap of DEGs of *tm3 stm3* and *q-ful* in VM, TM and FM/IM stages. Genes with p_adj_ < 0.01 in at least one stage were selected as DEG. (**b**) Expression of genes downstream of *tm3 stm3* and *q-ful* during reproductive meristem development. (**c**) *MBP13* and *MBP14* expression in VM, TM and FM/IM of *tm3 stm3* and *q-ful*. (**d**) *CKX4* and *CKX6* expression in VM, TM and FM/IM of *tm3 stm3* and *q-ful*. (**e**) Quantification of the primary transition until flowering of *tm3 stm3*, *mbp23 tm3 stm3 mbp18*, *ful2 mbp20* and *tm3 stm3 ful2 mbp20* under greenhouse conditions. (**f**) Expression of TFs downstream of *tm3 stm3* but not *q-ful* during reproductive meristem development. In (**b**–**d**,**f**), bars show mean FPKM values ± SEM. Asterisks indicate statistical significance according to the DESeq2 *P*_adj_ values from the mutant compared to its respective WT (* *P*_adj_ < 0.05, ** *P*_adj_ < 0.01, *** *P*_adj_ < 0.001). B3 domain TF, *Solyc01g108940*; AP2-like ERF, *Solyc06g068570*; Zinc finger TF, *Solyc06g065440*. In (**e**), different letters indicate statistically significant differences. FPKM, fragments per kilobase million; VM, vegetative meristem; TM, transition meristem; FM/IM, floral and inflorescence meristem.

To explain the delayed flowering in *tm3 stm3*, we performed an RNA-Seq experiment on VM, TM, and FM/IM tissues of WT and *tm3 stm3*. For each biological replicate, meristems from multiple plants at specific stages were harvested (as shown in Figure 1b) and pooled for RNA extraction. Expression of 288 marker genes for VM, TM, FM and IM tissue [38] showed a clear separation between the different meristematic tissues, indicating that the dissection of the different meristems was accurate (Supplementary Figure S5). The list of differentially expressed genes (DEGs) did not contain any obvious flowering regulator such as *SFT* or *FA*. Because the delayed flowering in *tm3 stm3* is very similar to the reported phenotypes of *ful2 mbp20* and *ful1 ful2 mbp10 mbp20* mutants (hereafter referred to as *q-ful* mutants, for ‘*quadruple ful*’) [8], and TM3 and STM3 can heterodimerize with the SlFULs (Figure 2a), we hypothesized that the TFs act together to regulate the floral transition. Overlapping the DEGs of *tm3 stm3* and *q-ful* meristems from all stages showed a significant enrichment for shared target genes (*P* = 2.2 × 10^−23^), in which 55 out of 328 DEGs of *tm3 stm3* overlap with *q-ful* targets (Figure 3a and Supplementary Table S1). As the majority of the shared targets (73%) is upregulated in both mutants, the putative SlSOC1/SlFUL complex probably acts predominantly as a repressive complex.

The large majority of overlapping DEGs showed a similar expression pattern in both the *tm3 stm3* and *q-ful* mutants. Among these DEGs were several TFs involved in plant development (Figure 3b and Supplementary Figure S7). For example, the tomato ortholog of *AHL15*, a gene that delays axillary meristem maturation in *Arabidopsis* [26], was significantly upregulated in both *tm3 stm3* and *q-ful*, suggesting that a complex of TM3/STM3/FUL2/MBP20 represses *AHL15* in the tomato apical meristems. The list of overlapping DEGs also contained two B3-domain TFs, including a putative close ortholog of *Arabidopsis REM16* according to Plaza5.0 [39]. Several B3-domain TFs, including *REM16*, have been linked to the vegetative-to-reproductive phase transition in *Arabidopsis* [40,41] and, possibly, this tomato B3-domain TF (*Solyc01g108940*) is also involved in flowering time regulation. Interestingly, the shared DEGs also included several *WRKY*s, which are usually associated with a response to environmental conditions, but have recently also been linked to regulating flowering time [42]. In particular, *WRKY28* was strongly upregulated in both mutants. Notably, we also found that the *SOC1*-like gene *MBP13* was upregulated in *tm3 stm3* and *q-ful*, and we observed the same trend for *MBP14*, together possibly alleviating the flowering phenotype of the mutants (Figure 3c). *AHL15* and *MBP13* have perfect CArG boxes in their promoters, and *MBP13* is bound by STM3 in the apex [28], supporting direct regulation by the *SOC1-* and *FUL*-like TFs.

Most interesting from the transcriptomic analysis was the overlap in cytokinin regulators as targets of *SlSOC1* and *SlFUL* genes. In *q-ful*, disturbed cytokinin signaling has been associated with its delayed flowering [8], and our RNA-Seq revealed that *CYTOKININ OXIDASE/DEHYDROGENASE 4* (*CKX4*) and *CKX6* were not only upregulated in *q-ful*, but also in *tm3 stm3* (Figure 3d). The increased expression of these cytokinin inhibitors suggests that in *tm3 stm3* the delay in flowering is also, at least partially, linked to disturbed cytokinin levels. Cytokinin enhances the activity of meristems, and thereby the rate of cell divisions and differentiation [43,44]. In line with expectations, we observed a slower development of the TM in both *tm3 stm3* and *ful2 mbp20* compared to WT (Supplementary Figure S6). The microscopic analyses of the meristems during the floral transition revealed that the delayed doming seemed to be caused both by a later initiation of differentiation towards the TM, and by a slower development of the TM itself.

### 2.4. TM3/STM3 Does Not Completely Depend on the SlFULs to Regulate the Floral Transition

To further investigate the genetic interaction between *TM3/STM3* and *FUL2/MBP20*, we studied the flowering time in *tm3 stm3 ful2 mbp20* mutants (Figure 3e). This quadruple mutant flowered even later than the *tm3 stm3*, *mbp23 tm3 stm3 mbp18*, or *ful2 mbp20* mutants, indicating that the *SlSOC1* and *SlFUL* genes do not act completely redundantly in the floral transition.

The additive effect of *TM3/STM3* and *FUL2/MBP20* on flowering time is also visible in the transcriptome data. In addition to the genes coregulated by *TM3/STM3* and the *SlFUL*s, we identified genes uniquely regulated by *TM3/STM3* (Figure 3f and Supplementary Figure S7). For example, expression of *AP2c* was increased in the VM of *tm3 stm3*, but not in *q-ful*, and the same trend was visible for its close homolog *AP2b*. It is probable that *AP2b* and *AP2c* contributed to the delayed flowering of *tm3 stm3*, as they are close homologs of *Arabidopsis AP2*-like floral repressors [45,46,47,48]. Most likely, STM3 directly inhibits *AP2b* and *AP2c*, as they were identified as STM3 targets in the apex by ChIP-Seq [28], and their promoters contain multiple imperfect but probably functional CArG boxes (e.g., CC[AT]_6_CG). Furthermore, some DEGs were weakly expressed in WT reproductive meristems and upregulated in *tm3 stm3*, suggesting that TM3/STM3 represses genes in the meristem that are functional in other tissues. Among others, an *AP2*-like *ERF* (*Solyc06g068570*) was strongly upregulated, which is involved in drought tolerance in leaves [49]. Other DEGs are expressed during reproductive meristem development and were increased in *tm3 stm3*, such as *CONSTANS* (*CO*) *interacting factor 2a*. Next to the association of this TF to drought tolerance [49], it may also play a role in flowering control by interacting with CO, a central player in the photoperiod flowering pathway [50]. We also identified several C2H2- and CCCH-type zinc finger TFs that were upregulated independently of the *SlFUL*s (a.o. *Solyc06g065440*), which are both classes of TFs with a broad role in plant development [51,52]. These expression data indicate that *TM3* and *STM3* are involved in an extensive network of TFs to regulate flowering time and probably other plant developmental processes. 

### 2.5. TM3/STM3 and FUL2/MBP20 Destabilize FM/IM Development, but in Opposite Ways

It has been reported that mutating *TM3* and *STM3* in the *j2 ej2* mutant background alleviates the branching phenotype of the *j2 ej2* mutant [7,22,28]. Interestingly, J2/EJ2 and FUL2/MBP20 most likely form a complex to regulate FM maturation and both double mutants show increased inflorescence branching [8,11]. Given that TM3/STM3 can heterodimerize with both J2/EJ2 and FUL2/MBP20 (Figure 2a), but appears to counteract the function of J2/EJ2 in the FM, we set out to elucidate the interactions between the MADS-domain TFs in reproductive meristem specification further. 

**Figure 4:**
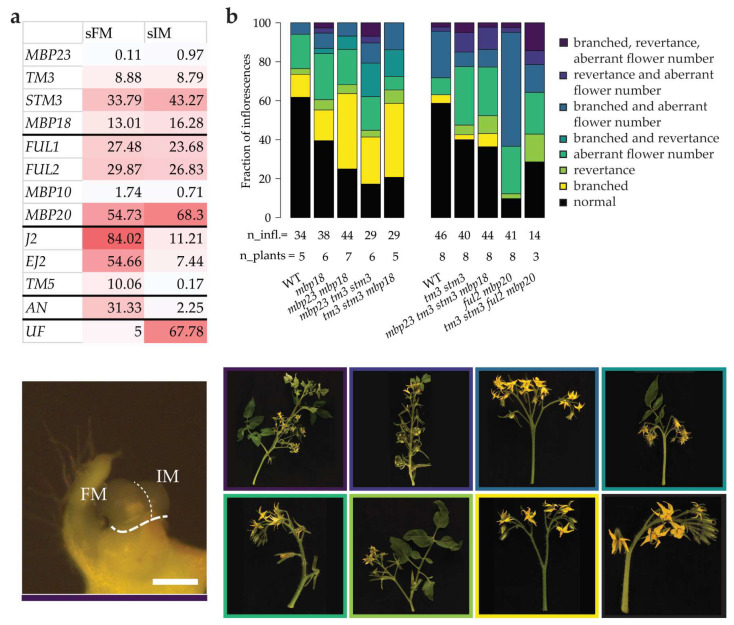
Functional characterization of *SlSOC1* and *SlFUL* genes in inflorescence development. (**a**) Expression of tomato *SOC1*, *FUL* and *SEP* homologs in the first sFM or sIM of WT plants. Shown are the average FPKM values of four biological replicates. *AN* and *UF* are shown as marker genes as a control for sampling stages. The stereomicroscope image at the bottom shows the harvested tissue. The scale bar is 200 µm. (**b**) Quantification of inflorescence phenotypes. A normal inflorescence was defined as unbranched, having no revertance to vegetative growth and 7 to 10 flowers. Inflorescences deviating from this were categorized and representative inflorescences are shown at the bottom with color-coded borders. The mutants were phenotyped in two independent screenings with separate WT controls, which had a consistent fraction of normal inflorescences.

To start, we studied the expression of the tomato *SOC1*, *FUL* and *SEP* homologs in Moneyberg WT sympodial FM (sFM) and sympodial IM (sIM) tissue separately (Figure 4a). As for the primary reproductive meristems, we pooled 20–50 meristems of visually the same stage per biological replicate prior to RNA extraction. The expression of *AN* and *UF*, marker genes for FM and IM identity, respectively [3,53], showed a clear separation of these meristems. The data revealed that the *SlSOC1* and *SlFUL* genes have similar expression patterns and are expressed in the sFM and sIM at equal levels. This suggests that the *SOC1* and *FUL* homologs play a role in both IM and FM development. On the contrary, *SEP*-like expression (*J2*, *EJ2*, *TM5*) was specific for the sFM and much lower in the sIM. *J2* and *EJ2* were especially highly expressed in the sFM, indicating that they are required for FM development and have a minor contribution to the establishment of IM cell fate.

To further unravel what the role is of *SlSOC1* and *SlFUL* genes in FM and IM specification, we phenotyped the inflorescences of single up to quadruple *slsoc1* mutants. We observed enormous variation in inflorescence traits, including branching, revertance to vegetative growth and flower number (Supplementary Figure S8). The large variation in phenotypes hindered the pinpointing of the exact result of the mutations, but indicated that the usually strict regulation of meristem development was disrupted in the mutants. To acquire a better understanding of the effects of the *tm3 stm3* mutations on the inflorescence phenotype, and investigate their effect on the increased branching phenotype of *ful2 mbp20*, we grew several mutant combinations in parallel. Categorizing the inflorescences on multiple traits revealed that inflorescence development was more disturbed in the *slsoc1* mutants than in WT (Figure 4b). In WT, 62% of inflorescences was normal, i.e., being unbranched, having no revertance to vegetative growth and 7 to 10 flowers. Inflorescences were quantified in two separate screenings, and while the fraction of abnormal inflorescences was consistent in WT, the type of destabilization differed over the screenings, indicating that the environment affects the type of destabilization. In the *slsoc1* mutants, inflorescence development was disturbed, as 17 to 40% of inflorescences was normal and the majority of inflorescences deviated from this standard by showing single or multiple deviating traits. 

To better understand the role of *TM3/STM3* in inflorescence development and their interaction with *FUL2/MBP20*, we compared the inflorescence phenotypes of the double and combinatorial quadruple mutants (Figure 4b). Inflorescence development was disturbed in both *tm3 stm3* and *ful2 mbp20*. Notably, these mutants showed distinct abnormal phenotypes. In *tm3 stm3*, the majority of abnormal inflorescences had less than 7 or more than 10 flowers without branching or revertance, and 18% of all inflorescences was branched. In *ful2 mbp20*, 58% of all inflorescences showed a typical phenotype that is branched and has more than 10 flowers. Combining these mutations in *tm3 stm3 ful2 mbp20* resulted in an alleviation of the *ful2 mbp20* branching phenotype, as the main category “branched and aberrant flower number” was strongly reduced from 58% to 14%. Furthermore, the proportion of normal inflorescences was increased to 33%, similar to *tm3 stm3*. These results indicate that *TM3/STM3* and *FUL2/MBP20* have opposite roles in inflorescence development.

## 3. Discussion

### 3.1. The Role of SlSOC1 Genes in the Floral Transition

Regulation of flowering time is crucial for plant reproductive success. The underlying gene regulatory network, including integration of flowering signals by *SOC1*, is largely conserved between the Brassicaceae and Asteraceae families [54]. Here, we showed that, also in tomato, several *SOC1* homologs have an evolutionarily conserved function in regulating flowering. The broad conservation of *SOC1*’s role in flowering regulation across angiosperms highlights the general importance of this gene. 

We identified four *SOC1* homologs in tomato that are expressed in the SAM upon the transition to flowering. CRISPR mutagenesis of these four genes revealed that *tm3* and *stm3* mutants display a significant delay in flowering in the indeterminate Moneyberg variety, which is in line with the previously reported role of *TM3* and *STM3* in the determinate cultivar M82 [7]. In addition to regulating the floral transition in the primary shoot, we show here that *TM3* and *STM3* also regulate the sympodial flowering time in Moneyberg. Our yeast protein–protein interaction data showed largely overlapping protein–protein interaction profiles for TM3 and STM3, suggesting that the paralogs act in a largely redundant manner. However, *STM3* contributes more strongly to the floral transition than *TM3*, most likely because its expression in the reproductive meristems is about tenfold higher, partially due to its gene duplication. Thus, TM3 and STM3 probably promote flowering initiation in a redundant, but dose-dependent manner.

Next to *TM3* and *STM3*, *MBP23* and *MBP18* are also related to *AtSOC1* and dynamically expressed during the floral transition. However, their mutants did not show strong flowering phenotypes. Flowering was mildly delayed in the *mbp18* single mutants, which only became clear in a growth chamber with a controlled environment. There was no visible flowering-time phenotype for *mbp23* mutants, nor did its mutation enhance the phenotype of the other mutants. It is possible, however, that *MBP23* is only induced under certain conditions that were not applied in our experiments. Another explanation for the mild or absent flowering phenotypes is a compensation effect of other *SOC1*-like proteins. For example, in *tm3 stm3*, the *SOC1*-like genes *MBP13* and *MBP14* were upregulated, possibly alleviating the flowering phenotype. A similar compensation effect by *SOC1*-like genes can explain the minor phenotypes in *mbp23* and *mbp18*. In *Arabidopsis*, the *SOC1*-like genes *AGL14*, *AGL19*, *AGL42*, *AGL71* and *AGL72* regulate the floral transition together with *SOC1* [30,55,56]. It is thus plausible that also in tomato, *SOC1*-like genes function partially redundantly and compensate for each other. Alternatively, *MBP23* and/or *MBP18* may have a more prominent role in other tissues. For example, the tomato eFP browser [57] shows that *MBP23* is most highly expressed in the roots, and may thus play a role in root development. Interestingly, *AtSOC1* is also expressed in the root, influences the number of lateral roots and is upregulated in response to nitrate deprivation [58]. A role for tomato *SOC1* homologs in root growth is therefore not unlikely. 

In *Arabidopsis*, *SOC1* is a true floral integrator gene, integrating signals from the different flowering pathways [12]. We found that in tomato, *TM3* and *STM3* clearly promote the transition to flowering, but they are not essential. The *tm3 stm3* mutants still flower, although later, and their phenotype is relatively mild compared to the severely delayed flowering phenotype of *atsoc1* [27]. Nevertheless, in all *slsoc1* mutants, we observed an increased variation in flowering time, which appeared to depend on the environmental conditions. This indicates that in the absence of TM3/STM3, the strict regulation of flowering time is lost and environmental pathways (e.g., light quality, temperature) become more pronounced. Possibly, the integrator function of *SOC1* is shared among multiple *SlSOC1* homologs, all responding to different signals, and together determining when the plant makes the transition to flowering, suggesting subfunctionalization within the *SlSOC1* clade. The upregulation of *MBP13* and *MBP14* in the *tm3 stm3* mutant may hint at this direction. 

### 3.2. SlSOC1 and SlFUL Proteins Interact during the Floral Transition

To explain the delayed flowering of *tm3 stm3*, we performed an RNA-Seq experiment of reproductive meristems. The major highlight of the transcriptomic analysis was the significant overlap in target genes of *TM3/STM3* and the *SlFUL*s, which included cytokinin regulators. Considering that TM3/STM3 can physically interact with FUL2/MBP20 (Figure 2a), that MADS-domain proteins often function in heteromeric complexes [20], and that all four TFs are important for the floral transition (Figure 3), it is likely that the upregulation of *CKX*s in *tm3 stm3* and *q-ful* is due to the alleviated binding of the tetramer TM3/STM3/FUL2/MBP20 to the *CKX4/6* promoters. TM3/FUL2 and TM3/MBP20 have already been shown in in vitro assays to bind as tetramers to the promoter of *CKX6* [8]. Since TM3 and STM3 have highly similar protein sequences, they are probably interchangeable in the tomato SOC1/FUL tetramer. Like *CKX6*, *CKX4* has a CArG box in its promoter, providing a binding site for the heterotetrameric MADS complex. These results suggest that the delayed flowering of *tm3 stm3* and *q-ful* is due to increased *CKX* expression, resulting in lower cytokinin levels, a less active SAM [43,44], and consequently a slower doming of the TM.

While *tm3 stm3* and *q-ful* are both delayed in flowering and share many target genes, the quadruple mutant *tm3 stm3 ful2 mbp20* is even more delayed in flowering (Figure 3e). This additive effect of mutations in *SlSOC1* and *SlFUL* indicates that these genes act partially, but not completely, redundantly, like in *Arabidopsis* [27]. Such independent functions of *SlSOC1* and *SlFUL* genes in the floral transition were further supported by the differential regulation of target genes between the mutants (Figure 3f and Supplementary Figure S7). For example, in *q-ful*, but not *tm3 stm3*, *CKX5* and *CKX8* were also upregulated, indicating that cytokinin regulation was more disturbed in *q-ful* than in *tm3 stm3*. On the other hand, other putative negative flowering regulators, including *AP2b* and *AP2c*, were more strongly upregulated in *tm3 stm3* than in *q-ful*. Several members of the *AP2*-like family in *Arabidopsis* function as floral repressors by promoting the vegetative phase [45,46,47,59]. In tomato, *TM3/STM3* are expressed earlier in the SAM than *FUL2/MBP20*, and may thus be more important for terminating vegetative fate via the repression of *AP2b/AP2c*. In line with this hypothesis, FUL2/MBP20 repress *AP2b/AP2c* later during secondary FM/IM development [8]. 

The subtle difference in flowering regulation between *tm3 stm3* and *q-ful* indicates that different MADS-domain protein complexes have varying DNA-binding affinities. Indeed, changes in binding specificity are a common mechanism underlying differential gene expression during development [60,61,62]. Next to the regulation of target genes by TM3/STM3-FUL2/MBP20, other MADS-domain protein complexes can be formed, especially when the native complex in the mutants is not functional anymore. TM3 and STM3 can also bind to the DNA as homodimers (Figure 2 and [8]). FUL2 and MBP20 cannot homodimerize and depend on interaction with other MADS-domain TFs, potentially J, which is highly expressed in reproductive meristems (Supplementary Figure S9) and can interact with FUL2 and MBP20 [8]. Therefore, it is likely that the tetrameric SlSOC1/SlFUL complex competes for binding with other MADS-domain protein complexes including tomato SOC1 or FUL homologs (e.g., MBP13, MBP14, FUL1), resulting in different transcriptomic changes in *tm3 stm3* and *q-ful*. Collectively, these changes in either mutant result in a similar cell identity shift and phenotype, namely, a delay of the floral transition.

### 3.3. SlSOC1 and SlFUL Genes Have Opposite Roles in Inflorescence Development

Besides the delayed floral transition, *tm3 stm3* and *ful2 mbp20* mutants also show an increase in abnormal inflorescences. When taking a closer look, the destabilization of inflorescence development in these mutants is clearly different. Interestingly, mutating *TM3* and *STM3* suppressed the inflorescence-branching phenotype of *ful2 mbp20*, suggesting that TM3/STM3 and FUL2/MBP20 have opposite roles in inflorescence development. TM3/STM3 have already been shown to act antagonistically to J2/EJ2 in inflorescence branching [7,22,28], and TM3/STM3 may have a similar role in counteracting FUL2/MBP20. FUL2/MBP20/J2/EJ2, together, seem to regulate inflorescence branching by promoting FM maturation, while TM3/STM3 have an opposite role in this process.

The question that remains is how TM3/STM3 can function antagonistically to FUL2/MBP20, although these four TFs seem to be spatiotemporally similarly expressed and can regulate target genes together. One possible explanation is that the resolution of our experiments was insufficient to capture spatial differences between TM3/STM3 and FUL2/MBP20. We successfully separated FM and IM tissue and *TM3*/*STM3* and *FUL2*/*MBP20* were similarly expressed in either meristem type. Still, there can be differences in expression in cell layers of these meristems, as is also the case for other MADS-domain proteins that give rise to the different floral organs by being active in specific cell layers [63]. So far, the transcriptomics of tomato meristems has not exceeded a single-meristem resolution [38]. A more detailed expression profile, either by transcriptional reporters or the novel stereo-seq [64], can answer whether *TM3*/*STM3* and *FUL2*/*MBP20* are truly co-expressed.

Another explanation why *TM3*/*STM3* and *FUL2*/*MBP20* have opposite effects on the developing inflorescence is that they compete in regulating target genes. FUL2/MBP20 and J2/EJ2 both promote FM maturation and can interact [8,10], and are thus likely to regulate targets together as a tetrameric complex. Alternatively, TM3/STM3 forms tetrameric complexes with either FUL2/MBP20 or J2/EJ2, thereby competing with the FM-promoting complex FUL2/MBP20/J2/EJ2. TM3/STM3, which can also bind to the DNA as homodimer [8], and therefore regulate targets independently. If TM3/STM3 interferes with the regulation of FUL2/MBP20/J2/EJ2 targets, this explains why TM3/STM3 and FUL2/MBP20 function antagonistically in inflorescence development. This hypothesis is supported by the observation that *STM3* and *J2* compete for binding at target genes [28]. However, overlapping *SlFUL* targets in the sFM/sIM [8] with *J2/EJ2* targets in the FM [10] did not provide promising candidates (e.g., B- and C-class flowering genes), possibly because slightly different tissues of different cultivars were compared.

### 3.4. The Role of SlSOC1 and SlFUL Genes in the Tomato Flowering Network

**Figure 5:**
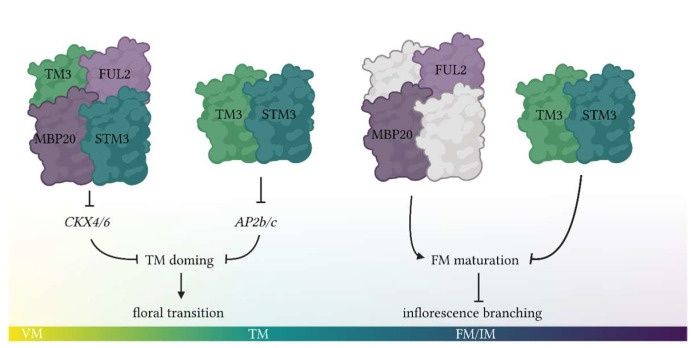
Model showing the proposed functions of SlSOC1 and SlFUL proteins in reproductive meristem development. White TFs interacting with FUL2/MBP20 are possibly J2/EJ2.

In this study, we investigated the function of *SlSOC1* genes in the floral transition and inflorescence development. We demonstrate that the tomato SOC1 homologs TM3 and STM3 interact with the FUL homologs FUL2 and MBP20 to promote the floral transition, while TM3/STM3 and FUL2/MBP20 probably have opposite roles in subsequent inflorescence development (Figure 5). Moreover, TM3/STM3 regulates target genes in either process independently of the SlFULs, thereby adding an additional layer of floral activation to the TM, and allowing this complex to act antagonistically of the SlFULs in FM/IM development. While several open questions remain that will need to be addressed in other studies, this work resolves a piece of the puzzle of reproductive meristem development in tomato.

## 4. Materials and Methods

### 4.1. Plant Materials and Growing Conditions

*Solanum lycopersicum* L. cultivar Moneyberg was used as the WT. Plants were grown on rockwool blocks under a 16 h light/8 h dark cycle. Seedlings were grown in a growth chamber at 21 °C and watered with 1 g/L hyponex solution. After 5–8 weeks, the plants were transferred to a greenhouse, watered by fertigation and grown under natural light supplemented with sodium lights. The mutants *ful2 mbp20* and *q-ful* were obtained from Jiang et al. [8], and *tm3 stm3 ful2 mbp20* was obtained by crossing *tm3 stm3 mbp18* with *ful2 mbp20* and selecting F2 homozygous *tm3 stm3 ful2 mbp20* mutants.

### 4.2. Phylogenetic Analysis of SOC1 Homologs

For phylogenetic analysis, 69 amino acid sequences from *SOC1*-like proteins were retrieved from the Sol Genomics Network [65] and the National Center for Biotechnology Information [66]. All amino acid sequences are listed in Supplementary Table S2. Sequences were analyzed in MEGA X 10.1.7 [67] and aligned with the MUSCLE algorithm, using default settings [68]. Evolutionary history was inferred using the maximum likelihood method and JTT matrix-based model [69]. The bootstrap consensus tree [70] inferred from 5000 replicates was taken to represent the evolutionary history of the analyzed taxa. Branches were collapsed if they corresponded to partitions in less than 50% of bootstrap replicates. Initial trees for the heuristic search were obtained automatically by applying Neighbour-Join and BioNJ algorithms to a matrix of pairwise distances, which were estimated using a JTT model. The phylogenetic tree giving the superior log likelihood value was selected. All positions with less than 95% site coverage were eliminated, i.e., fewer than 5% alignment gaps, missing data, and ambiguous bases were allowed at any position (partial deletion option). 

### 4.3. Meristem Imaging

Meristem images were taken with a stereomicroscope (Stemi 508, Zeiss, Oberkochen, Germany) coupled to a camera (Axiocam 105 color, Zeiss, Oberkochen, Germany). Prior to imaging, the meristems were exposed by removing older leaves using forceps. 

### 4.4. Meristem Transcriptome Profiling by RT-qPCR and RNA-Seq

For gene expression analysis, RNA was isolated from the VM, TM and FM/IM (primary reproductive meristems) or separate sFM and sIM (reproductive meristem of first sympodial unit). Meristems were hand-dissected under a stereomicroscope, directly frozen in liquid nitrogen, and RNA was extracted using the Arcturus PicoPure RNA Extraction kit (ThermoFisher Scientific, Waltham, MA, USA). Primary reproductive meristems were sampled in triplicate and sFM and sIM in quadruplicate, meaning that three and four independent batches of WT and mutant plants were grown, respectively. For RNA extraction, 11 to 52 meristems of the same stage were pooled per biological replicate, yielding 0.27 to 1.43 µg RNA. The VM was sampled just before the visible doming of the meristem. The TM was harvested when the meristem had domed, but had not bifurcated yet. The first formed FM and IM of the inflorescence were sampled before the visible formation of flower organs, either together for primary FM/IM samples, or separately for sFM and sIM samples. 

For qPCR analysis, DNA was removed from the samples using the TURBO DNA-free Kit (ThermoFisher Scientific, MA, USA). cDNA was synthesized using the iScript cDNA synthesis kit (BioRad, Hercules, CA, USA). Real-time qPCR was performed with iQ SYBR Green Supermix (BioRad, CA, USA) in a CFX Connect Real-Time machine (BioRad, CA, USA). *CAC* was used as reference gene. All used primers are listed in Supplementary Table S3.

For the RNA-seq, library preparation and sequencing were performed at BGI Genomics. RNA quality was validated using the Agilent 2100 Bioanalyzer and transcripts were sequenced using 2 × 150 bp paired-end DNB Sequencing. Adapter sequences, contamination and low-quality reads were removed from the raw data. Filtered reads were mapped to the tomato SL4.0 genome using HISAT2 [71] with default parameters. Counts per gene were generated using StringTie in expression-estimation mode [72] (Supplementary Table S4). DEGs were calculated using DESeq2 [73], while only considering genes with at least 10 read counts (Supplementary Table S1). A hypergeometric test was used to calculate the significance of the overlapping DEGs between datasets. 

The raw data from the two RNA-Seq experiments are publicly available in the Sequence Read Archive (SRA, ncbi.nlm.nih.gov/sra) with the accession number PRJNA980935.

### 4.5. Yeast Two-Hybrid (Y2H)

To study protein–protein interactions, a Y2H screening using the GAL4 system was performed according to De Folter and Immink [74]. The full-length coding sequences were amplified from IM cDNA and cloned into pDEST32 and pDEST22 vectors, respectively, for bait and prey proteins. The expression vectors were transformed into the yeast strains PJ69-A (pDEST22) and PJ69-4α (pDEST32). Protein–protein interaction was screened on -LWH dropout medium containing 3 mM 3-amino-1,2,4-triazole (3-AT), or on -LWA dropout medium. Plates were incubated at 20 °C for five days. The screening was performed twice in both reciprocal directions (AD × BD and BD × AD). All primers used for cloning are listed in Supplementary Table S3.

### 4.6. CRISPR Construct Generation and Stable Tomato Transformation

CRISPR sgRNAs were designed on CRISPOR [75]. CRISPR constructs were assembled using the MoClo toolkit and Golden Gate cloning [76,77]. Each sgRNA was expressed under an *Arabidopsis* U6-26 promoter, and hCas9 was driven by 2 × proCaMV35S as described by Slaman et al. [78]. *35S::GFP* and *NOS::NPTII* were used as selection markers. All primers used for cloning are listed in Supplementary Table S3.

*Agrobacterium tumefaciens* strain C58C1 was used for stable tomato transformations according to Van Roekel et al. [79], except that 2 mg/L zeatin was used instead of zeatin riboside, and that medium B did not contain IAA, but was supplemented with 0.05 mg/L 2,4-D. Explants were cultured in a growth chamber at 25 °C with 16 h light, at an intensity of 60 µE at plate level, and 8 h dark. Transgenic seedlings were tested for ploidy with Iribov Analytical Services B.V. Diploid plants with deleterious mutations according to PROVEAN [80] due to a frameshift leading to a premature stop codon or due to a deletion of the essential amino acids that were selected.

### 4.7. Phenotyping

Flowering time was phenotyped in the greenhouse in four independent screenings in spring or winter, or in a growth chamber. The primary floral transition was determined by counting the number of leaves until the primary inflorescence, the sympodial flowering time by the number of leaves in at least five SUs. The average size of the sympodial units was used to test the statistical significance. Inflorescence architecture was scored in completely developed inflorescences. Statistical significance was determined by one-way ANOVA and Tukey post-test (α = 0.05). Per genotype, two to five transgenic lines from independent transformations with independent mutations were phenotyped. Only for *mbp23*, one independent transgenic line was obtained, with two different homozygous alleles in the T2 generation.

### 4.8. Accession Numbers

AHL15, Solyc12g087950; AP2b, Solyc02g064960; AP2c, Solyc02g093150; AP2-like ERF, Solyc06g068570; B3 domain TF, Solyc01g108940; B3 domain TF (2), Solyc01g108930; CKX4, Solyc04g080820; CKX5, Solyc04g016430; CKX6, Solyc12g008900; CKX8, Solyc10g017990; CO-interacting protein 2a, Solyc06g072040; EJ2 (MADS1), Solyc03g114840; ERF1a, Solyc04g014530; ERF2, Solyc11g011740; FUL1, Solyc06g069430; FUL2, Solyc03g114830; J, Solyc11g010570; J2 (MBP21), Solyc12g038510; MADS-RIN, Solyc05g012020; MBP10, Solyc02g065730; MBP12, Solyc12g088090; MBP13, Solyc08g080100; MBP14, Solyc12g056460; MBP18 (FYFL), Solyc03g006830; MBP19, Solyc06g035570; MBP20, Solyc02g089210; MBP22, Solyc11g005120; MBP23, Solyc10g01640/30; MBP24, Solyc01g105800; MBP9, Solyc04g076680; MC, Solyc05g056620; STM3, Solyc01g092950; TAG1, Solyc02g071730; TM3, Solyc01g160330/Solyc01g093965; TM5, Solyc05g015750; WRKY28, Solyc12g011200; WRKY44, Solyc10g084380; Zinc finger TF, Solyc06g065440; Zinc finger TF 34, Solyc04g064770.

## Data Availability

The data supporting the results presented in this article can be found in the main figures and Supplementary tables and figures. The raw data from the two RNA-Seq experiments are publicly available in the Sequence Read Archive (SRA, ncbi.nlm.nih.gov/sra) with the accession number PRJNA980935.

## Supplementary Materials

The following supporting information can be downloaded at https://www.mdpi.com/article/10.3390/plants12152754/s1. Figure S1: Sequence characteristics of *SOC1*-like genes and proteins; Figure S2: Y2H screening showing interactions of SOC1-like proteins with several MADS-domain TFs; Figure S3: Mutations of *SlSOC1* genes generated by CRISPR/Cas9 mutagenesis; Figure S4: Quantification of the primary transition to flowering in *slsoc1* mutants under greenhouse conditions; Figure S5: Scaled expression of 288 marker genes for VM, TM and FM stages identified by Meir et al. [38] in WT and *tm3 stm3* RNA-Seq samples; Figure S6: Stereomicroscope images of reproductive meristem development in WT, *tm3 stm3* and *ful2 mbp20*; Figure S7: Expression of several genes of interest in VM, TM and FM/IM of *tm3 stm3* and *q-ful*; Figure S8: Quantification of inflorescence traits in several *slsoc1* and *ful2 mbp20* mutants; Figure S9: Expression of *J* during reproductive meristem development; Table S1: Overview of DEGs of RNA-Seq data; Table S2: Protein sequences used to build the phylogenetic tree in Figure 1a; Table S3: List of primers; Table S4: Overview of read counts of RNA-Seq data.
