# Supplementary material for: TM3 and STM3 Promote Flowering Together with FUL2 and MBP20, but Act Antagonistically in Inflorescence Branching in Tomato
Source: Plants (Basel). 2023 Jul 25;12(15):2754. doi: 10.3390/plants12152754 (PMC10420972; doi:10.3390/plants12152754)
Supplement: Supplementary file 1 [file plants-12-02754-s001.zip › Supplementary Figures.pdf]

List of Supplementary Materials:

Figures

1. Structure of the *TM3*/*STM3* locus and alignment of four SOC1-like proteins.
2. Yeast two-hybrid raw data.
3. Genotypes of CRISPR mutants.
4. Flowering time data separated per screening, including single mutants.
5. Marker genes of VM/TM/FM in RNA-Seq samples of the primary inflorescence.
6. Stereomicroscope images of reproductive meristem development in WT, *tm3 stm3* and *ful2 mbp20*
7. Additional FPKM plots, not shown in Figure 3.
8. Separate figures of branching, revertance and flower numer.
9. Expression of *J* in reproductive meristems.

Tables

1. Overview of RNA-Seq data, DEGs.
2. Protein sequences to build the phylogenetic tree in Figure 1a.
3. Primers used in this study.
4. Overview of RNA-Seq data, read counts.

a

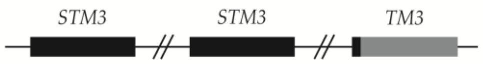

b

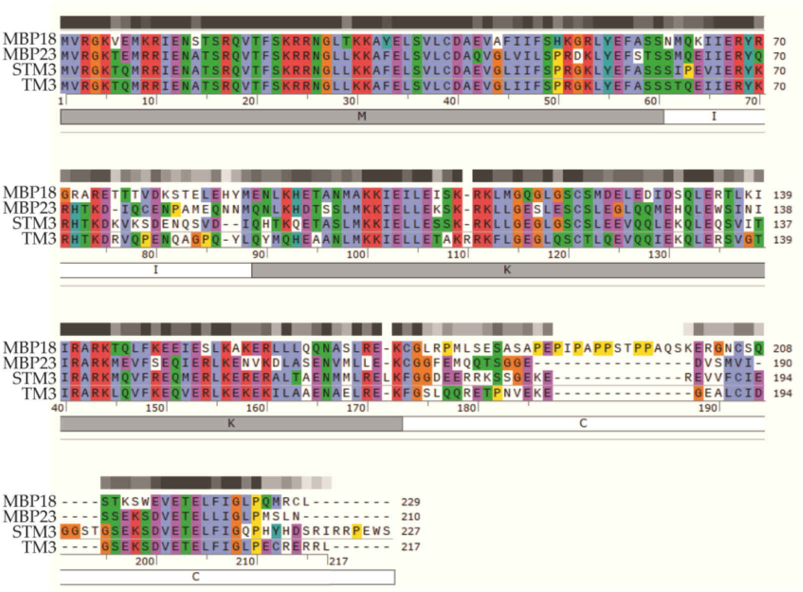

Supplementary Figure S1: Sequence characteristics of *SOC1*-like genes and proteins. (**a**) Structure of the *TM3*/*STM3* locus. *TM3* and *STM3* are located in tandem, with *STM3* having two identical copies. The first exon of *TM3* is identical to the first exon of *STM3*. (**b**) MUSCLE alignment of MBP18, MBP23, STM3 and TM3. Alternating grey and white blocks show MIKC domains. Color highlighting is based on properties and conservation (Clustal X). Grey blocks on top of the alignment show sequence conservation.

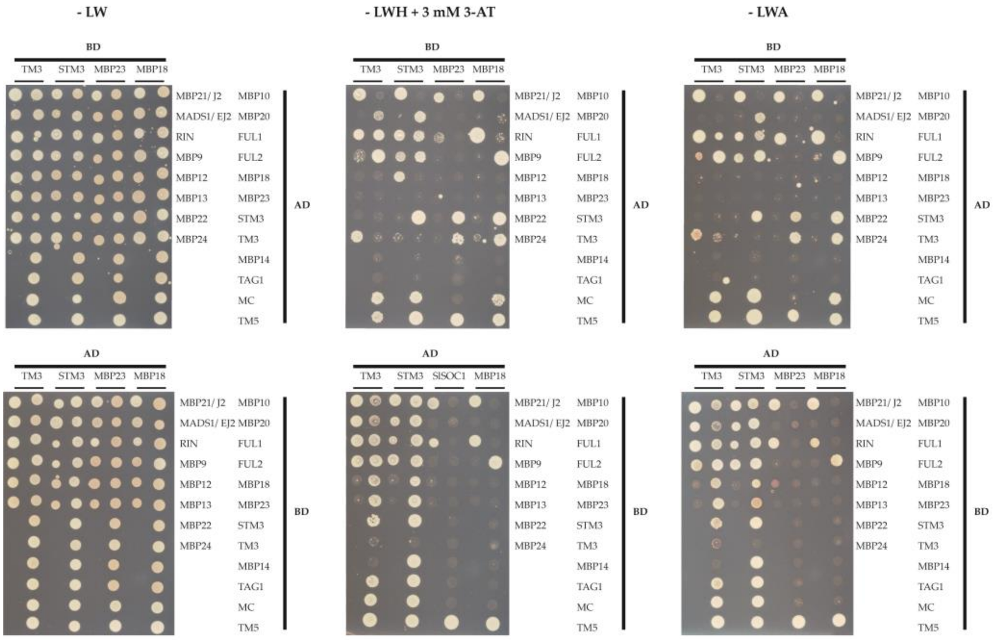

Supplementary Figure S2: Y2H screening showing interactions of SOC1-like proteins with several MADS-domain TFs. Top: SOC1-like proteins as bait (fused to BD). Bottom: SOC1-like proteins as prey (fused to AD). BD, binding domain; AD, activation domain; L, leucine; W, tryptophan; H, histidine; A, adenine; 3-AT, 3-amino-1,2,4-triazole.

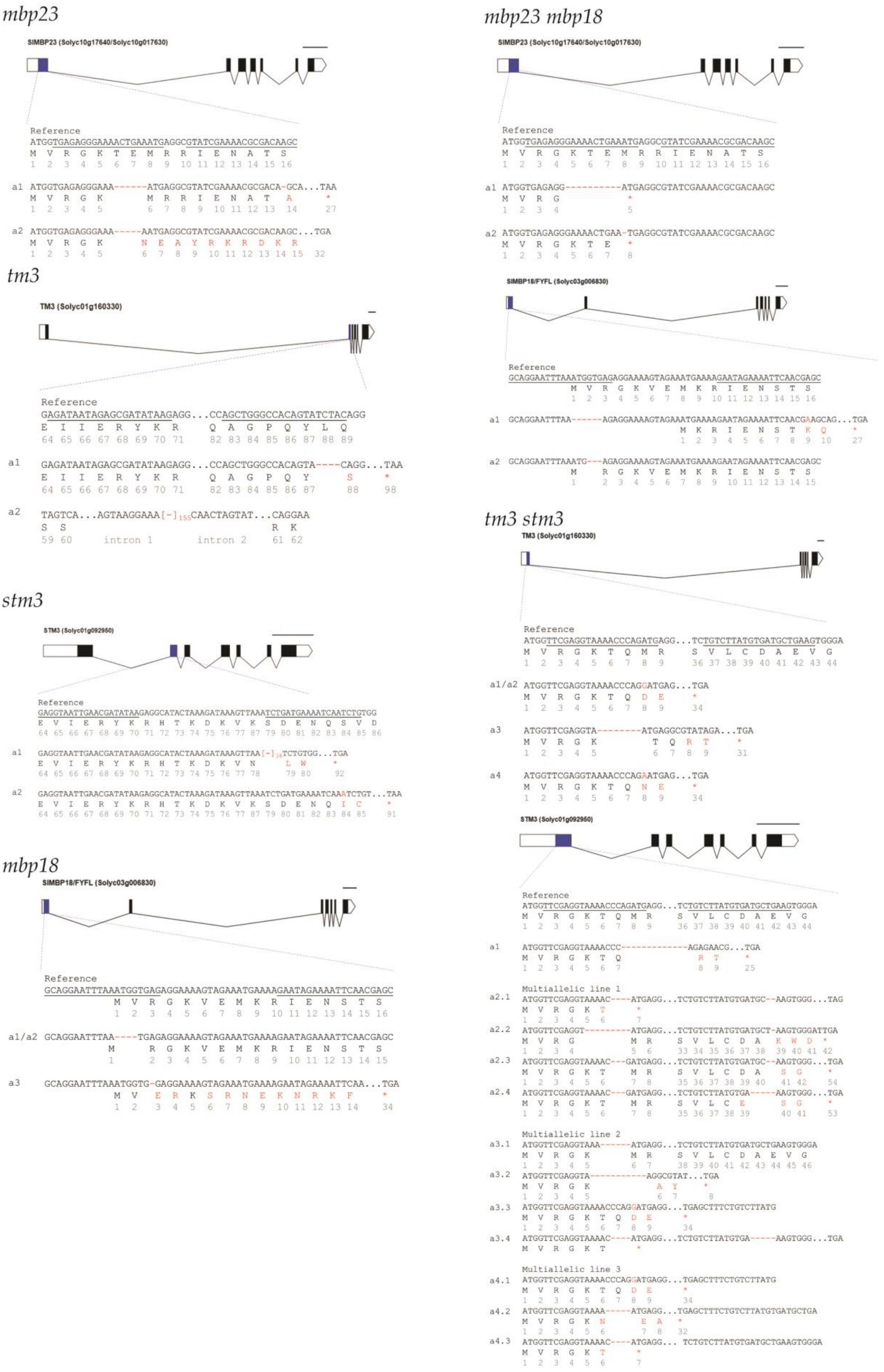

mbp23
SlMBP23 (Solyc10g17640/Solyc10g017630)
Reference
ATGGTGAGAGGGAAAACTGAAATGAGGCGTATCGAAAACGCGACAAGC
M V R G K T E M R R I E N A T S
1 2 3 4 5 6 7 8 9 10 11 12 13 14 15 16
a1 ATGGTGAGAGGGAAA------ATGAGGCGTATCGAAAACGCGACA-GCA...TAA
M V R G K M R R I E N A T A *
1 2 3 4 5 6 7 8 9 10 11 12 13 14 27
a2 ATGGTGAGAGGGAAA-----AATGAGGCGTATCGAAAACGCGACAAGC...TGA
M V R G K N E A Y R K R D K R
1 2 3 4 5 6 7 8 9 10 11 12 13 14 15 32
tm3
TM3 (Solyc01g160330)
Reference
GAGATAATAGAGCGATATAAGAGG...CCAGCTGGGCCACAGTATCTACAGG
E I I E R Y K R Q A G P Q Y L Q
64 65 66 67 68 69 70 71 82 83 84 85 86 87 88 89
a1 GAGATAATAGAGCGATATAAGAGG...CCAGCTGGGCCACAGTA----CAGG...TAA
E I I E R Y K R Q A G P Q Y S *
64 65 66 67 68 69 70 71 82 83 84 85 86 87 88 98
a2 TAGTCA...AGTAAGGAAA[-]155CAACTAGTAT...CAGGAA
S S R K
59 60 intron 1 intron 2 61 62
stm3
STM3 (Solyc01g092950)
Reference
GAGGTAATTGAACGATATAAGAGGCATACTAAAGATAAAGTTAAATCTGATGAAAATCAATCTGTGG
E V I E R Y K R H T K D K V K S D E N Q S V D
64 65 66 67 68 69 70 71 72 73 74 75 76 77 87 79 80 81 82 83 84 85 86
a1 GAGGTAATTGAACGATATAAGAGGCATACTAAAGATAAAGTTAA[-]16TCTGTGG...TGA
E V I E R Y K R H T K D K V N L W *
64 65 66 67 68 69 70 71 72 73 74 75 76 77 78 79 80 92
a2 GAGGTAATTGAACGATATAAGAGGCATACTAAAGATAAAGTTAAATCTGATGAAAATCAAATCTGT...TAA
E V I E R Y K R H T K D K V K S D E N Q I C *
64 65 66 67 68 69 70 71 72 73 74 75 76 77 87 79 80 81 82 83 84 85 91
mbp18
SlMBP18/FYFL (Solyc03g006830)
Reference
GCAGGAATTTAAATGGTGAGAGGAAAAGTAGAAATGAAAAGAATAGAAAATTCAACGAGC
M V R G K V E M K R I E N S T S
1 2 3 4 5 6 7 8 9 10 11 12 13 14 15 16
a1/a2 GCAGGAATTTAA----TGAGAGGAAAAGTAGAAATGAAAAGAATAGAAAATTCAACGAGC
M R G K V E M K R I E N S T S
1 2 3 4 5 6 7 8 9 10 11 12 13 14 15
a3 GCAGGAATTTAAATGGTG-GAGGAAAAGTAGAAATGAAAAGAATAGAAAATTCAA...TGA
M V E R K S R N E K N R K F *
1 2 3 4 5 6 7 8 9 10 11 12 13 14 34
mbp23 mbp18
SlMBP23 (Solyc10g17640/Solyc10g017630)
Reference
ATGGTGAGAGGGAAAACTGAAATGAGGCGTATCGAAAACGCGACAAGC
M V R G K T E M R R I E N A T S
1 2 3 4 5 6 7 8 9 10 11 12 13 14 15 16
a1 ATGGTGAGAGG----------ATGAGGCGTATCGAAAACGCGACAAGC
M V R G *
1 2 3 4 5
a2 ATGGTGAGAGGGAAAACTGAA-TGAGGCGTATCGAAAACGCGACAAGC
M V R G K T E *
1 2 3 4 5 6 7 8
SlMBP18/FYFL (Solyc03g006830)
Reference
GCAGGAATTTAAATGGTGAGAGGAAAAGTAGAAATGAAAAGAATAGAAAATTCAACGAGC
M V R G K V E M K R I E N S T S
1 2 3 4 5 6 7 8 9 10 11 12 13 14 15 16
a1 GCAGGAATTTAA------AGAGGAAAAGTAGAAATGAAAAGAATAGAAAATTCAACGAAGCAG...TGA
M K R I E N S T K Q *
1 2 3 4 5 6 7 8 9 10 27
a2 GCAGGAATTTAAATG---AGAGGAAAAGTAGAAATGAAAAGAATAGAAAATTCAACGAGC
M R G K V E M K R I E N S T S
1 2 3 4 5 6 7 8 9 10 11 12 13 14 15
tm3 stm3
TM3 (Solyc01g160330)
Reference
ATGGTTCGAGGTAAAACCCAGATGAGG...TCTGTCTTATGTGATGCTGAAGTGGGA
M V R G K T Q M R S V L C D A E V G
1 2 3 4 5 6 7 8 9 36 37 38 39 40 41 42 43 44
a1/a2 ATGGTTCGAGGTAAAACCCAGGATGAG...TGA
M V R G K T Q D E *
1 2 3 4 5 6 7 8 9 34
a3 ATGGTTCGAGGTA--------ATGAGGCGTATAGA...TGA
M V R G K T Q R T *
1 2 3 4 5 6 7 8 9 31
a4 ATGGTTCGAGGTAAAACCCAGAATGAG...TGA
M V R G K T Q N E *
1 2 3 4 5 6 7 8 9 34
STM3 (Solyc01g092950)
Reference
ATGGTTCGAGGTAAAACCCAGATGAGG...TCTGTCTTATGTGATGCTGAAGTGGGA
M V R G K T Q M R S V L C D A E V G
1 2 3 4 5 6 7 8 9 36 37 38 39 40 41 42 43 44
a1 ATGGTTCGAGGTAAAACCC-------------AGAGAACG...TGA
M V R G K T Q R T *
1 2 3 4 5 6 7 8 9 25
Multiallelic line 1
a2.1 ATGGTTCGAGGTAAAAC----ATGAGG...TCTGTCTTATGTGATGC--AAGTGGG...TAG
M V R G K T *
1 2 3 4 5 6 7
a2.2 ATGGTTCGAGGT---------ATGAGG...TCTGTCTTATGTGATGCT-AAGTGGGATTGA
M V R G M R S V L C D A K W D *
1 2 3 4 5 6 33 34 35 36 37 38 39 40 41 42
a2.3 ATGGTTCGAGGTAAAAC---GATGAGG...TCTGTCTTATGTGATGC--AAGTGGG...TGA
M V R G K T M R S V L C D A S G *
1 2 3 4 5 6 7 8 35 36 37 38 39 40 41 42 54
a2.4 ATGGTTCGAGGTAAAAC---GATGAGG...TCTGTCTTATGTGA-----AAGTGGG...TGA
M V R G K T M R S V L C E S G *
1 2 3 4 5 6 7 8 35 36 37 38 39 40 41 53
Multiallelic line 2
a3.1 ATGGTTCGAGGTAAA------ATGAGG...TCTGTCTTATGTGATGCTGAAGTGGGA
M V R G K M R S V L C D A E V G
1 2 3 4 5 6 7 38 39 40 41 42 43 44 45 46
a3.2 ATGGTTCGAGGTA-----------AGGCGTAT...TGA
M V R G K A Y *
1 2 3 4 5 6 7 8
a3.3 ATGGTTCGAGGTAAAACCCAGGATGAGG...TGAGCTTTCTGTCTTATG
M V R G K T Q D E *
1 2 3 4 5 6 7 8 9 34
a3.4 ATGGTTCGAGGTAAAAC----ATGAGG...TCTGTCTTATGTGA-----AAGTGGG...TGA
M V R G K T *
Multiallelic line 3
a4.1 ATGGTTCGAGGTAAAACCCAGGATGAGG...TGAGCTTTCTGTCTTATG
M V R G K T Q D E *
1 2 3 4 5 6 7 8 9 34
a4.2 ATGGTTCGAGGTAAAA-----ATGAGG...TGAGCTTTCTGTCTTATGTGATGCTGA
M V R G K N E A *
1 2 3 4 5 6 7 8 32
a4.3 ATGGTTCGAGGTAAAAC----ATGAGG... TCTGTCTTATGTGATGCTGAAGTGGGA
M V R G K T *
1 2 3 4 5 6 7

mbp23 tm3 stm3

SlMBP23 (Solyc10g17640/Solyc10g017630)

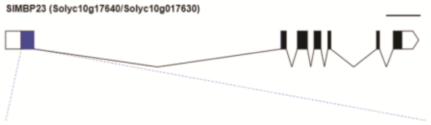

```
   Reference
   ATGGTGAGAGGGAAAACTGAAATGAGGCGTATCGAAAACGCGACAAGC
   M  V  R  G  K  T  E  M  R  R  I  E  N  A  T  S
   1  2  3  4  5  6  7  8  9  10 11 12 13 14 15 16

a1 ATGGTGAGAGGGAAAACTGAAATGAGGCGTATCGAAAAC-------GCAGGC...TAA
   M  V  R  G  K  T  E  M  R  R  I  E  N        A  G     *
   1  2  3  4  5  6  7  8  9  10 11 12 13       14 15    27

a2 ATGGTGAGAGGGA------AAATGAGGCGTATCGAAAACG-----AGCAGGCA...TGA
   M  V  R  G  K        M  R  R  I  E  N  E      Q  A     *
   1  2  3  4  5        6  7  8  9  10 11 12     13 14    30

a3 ATGGTGAGAGGGAAA----AAATGAGGCGTATCGAAAACGCGACAAGC
   M  V  R  G  K        K  *
   1  2  3  4  5        6  7
```

TM3 (Solyc01g160330)

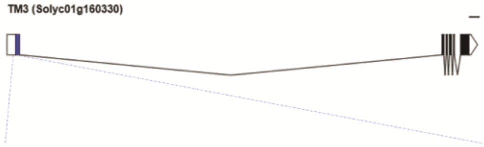

```
   Reference
   ATGGTTCGAGGTAAAACCCAGATGAGG...TCTGTCTTATGTGATGCTGAAGTGGGA
   M  V  R  G  K  T  Q  M  R     S  V  L  C  D  A  E  V  G
   1  2  3  4  5  6  7  8  9     36 37 38 39 40 41 42 43 44

a1 ATGGTTCGAGGTA---------ATGAGGCG...TGAGCTTTCTGTCTTATGTGATGCT
   M  V  R  G  N           E  A      *
   1  2  3  4  5           6  7      31

a2 ATGGTTCGAGGTAAAACCCAGGATGAG...TGAGCTTTCTGTCTTATGTGATGCT
   M  V  R  G  K  T  Q  D  E     *
   1  2  3  4  5  6  7  8  9     34

a3 ATGGTTCGAGGTAAAACCCAG[-]_103 AAGTGGGATTGA
   M  V  R  G  K  T  Q         K  W  D  *
   1  2  3  4  5  6  7         8  9  10 11
```

STM3 (Solyc01g092950)

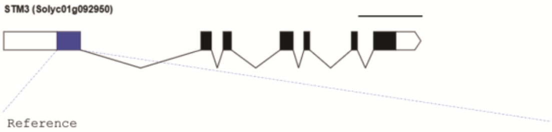

```
   Reference
   ATGGTTCGAGGTAAAACCCAGATGAGG...TCTGTCTTATGTGATGCTGAAGTGGGA
   M  V  R  G  K  T  Q  M  R     S  V  L  C  D  A  E  V  G
   1  2  3  4  5  6  7  8  9     36 37 38 39 40 41 42 43 44

a1 ATGGTTCGAGGTAAAA--------AGGCGTAT...TGA
   M  V  R  G  K  K          A  Y     *
   1  2  3  4  5  6          7  8     31

a2 ATGGTTCGAGGTAAAAC----ATGAGG...TCTGTCTTATGTGATGCTGAAAGTGGGA
   M  V  R  G  K  T        *
   1  2  3  4  5  6        7

a3 ATGGTTCGAGGTAAAACCCAGGATGAGG...TGAGCTTTCTGTCTTATGTGATGCTGAAGTGGG
   M  V  R  G  K  T  Q  D  E        *
   1  2  3  4  5  6  7  8  9        34
```

tm3 stm3 mbp18

TM3 (Solyc01g160330)

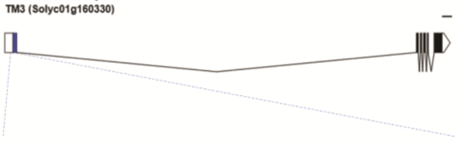

```
     Reference
     ATGGTTCGAGGTAAAACCCAGATGAGG...TCTGTCTTATGTGATGCTGAAGTGGGA
     M  V  R  G  K  T  Q  M  R     S  V  L  C  D  A  E  V  G
     1  2  3  4  5  6  7  8  9     36 37 38 39 40 41 42 43 44

a1   ATGGTTCGAGGTA-------GATGAGG...TCTGTCTTATGTGATGCTGAAGTGGGA
     M  V  R  G  R         *
     1  2  3  4  5         6

a2   ATGGTTCGAGGTAAAACCCAG[-]_15[insertion]_88
     M  V  R  G  K  T  Q        N  A  S  F  *
     1  2  3  4  5  6  7        8  9  61 62 63

a3   ATGGTTCGAGGTAAAACCCAGAATGAG...TGA
     M  V  R  G  K  T  Q  N  E     *
     1  2  3  4  5  6  7  8  9     34

a4   ATGGTTCGAGGTAAAA---------------TA...TCTGTCTTATGTGATGCTGTAAGT...TAA
     M  V  R  G  K  I                    S  V  L  C  D  A  V  S     *
     1  2  3  4  5  6                    31 32 33 34 35 36 37 38    55
```

STM3 (Solyc01g092950)

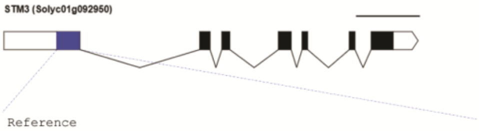

```
     Reference
     ATGGTTCGAGGTAAAACCCAGATGAGG...TCTGTCTTATGTGATGCTGAAGTGGGA
     M  V  R  G  K  T  Q  M  R     S  V  L  C  D  A  E  V  G
     1  2  3  4  5  6  7  8  9     36 37 38 39 40 41 42 43 44

a1   ATGGTTCGAGGTAA-------ATGAGG...TCTGTCTTATGTGATGCTGAAGTGGGA
     M  V  R  G  K           *
     1  2  3  4  5           6

a2   ATGGTTCGAGGTA------AGATGAGG...TCTGTCTTATGTGATGCTGAAGTGGGA
     M  V  R  G  K          M  R     S  V  L  C  D  A  E  V  G
     1  2  3  4  5          6  7     34 35 36 37 38 39 40 41 42

a3   ATGGTTCGAGGTAAAACCCAGGATGAGG...TGAGCTTTCTGTCTTATGTGATGCTGAAGTGGG
     M  V  R  G  K  T  Q  D  E        *
     1  2  3  4  5  6  7  8  9        34

a4   ATGGTTCGAGGTAAAA-----ATGAGG...TGAGCTTTCTGTCTTATGTGATGCTGAAGTGGGA
     M  V  R  G  K  N        E  A     *
     1  2  3  4  5  6        7  8     32

     Multiallelic line 1
a5.1 ATGGTTCGAGGTAAAACCCAGGATGAGG...TGAGCTTTCTGTCTTATGTGATGCTGAAGTGGG
     M  V  R  G  K  T  Q  D  E        *
     1  2  3  4  5  6  7  8  9        34
a5.2 ATGGTTCGAGGTAAAACCCAGGATGAGG...TGAGCTTTCTGTCTTATGTGATGCTGTAAGT...TGA
     M  V  R  G  K  T  Q  D  E        *
     1  2  3  4  5  6  7  8  9        34
a5.3 ATGGTTCGAGGTA--------ATGAGG...TGAGCTTTCTGTCTTATGTGATGCTGAAGTGGGA
     M  V  R  G  N           E  A     *
     1  2  3  4  5           6  7     31
a5.4 ATGGTTCGAGGTA--------ATGAGG...TGAGCTTTCTGTCTTATGTGAT--TGAAGTGGGA
     M  V  R  G  N           E  A     *
     1  2  3  4  5           6  7     31
```

SlMBP18/FYFL (Solyc03g006830)

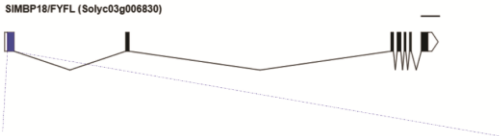

```
     Reference
     GCAGGAATTTAAATGGTGAGAGGAAAAGTAGAAATGAAAAGAATAGAAAATTCAACGAGC
                 M  V  R  G  K  V  E  M  K  R  I  E  N  S  T  S
                 1  2  3  4  5  6  7  8  9  10 11 12 13 14 15 16

a1   GCAGGAATTTAAATG--GAGAGGAAAAGTAGAAATGAAAAG...TGAAAATTCAACGAGC
                 M     E  R  K  S  R  N  E  K     *
                 1     2  3  4  5  6  7  8  9     33

a2   GCAGGAATTTAAATGGTTGAGAGGAAAAGTAGAAATGAAAAG...TGAAAATTCAACGAGC
                 M  V  E  R  K  S  R  N  E  K     *
                 1  2  3  4  5  6  7  8  9  10    34

a3   GCAGGAATTTA-----TGAGAGGA...AGAATAGAAAATTCAACGAAGCAG...TGA
               M        R  G     R  I  E  N  A  T  K  Q     *
               1        2  3     9  10 11 12 13 14 15 16    33

a4   GCAGGAATTTAAA----GAGAGGAAAAGTAGAAATGAAAAGAATAGAAAATTCAACGAGC
                                    M  K  R  I  E  N  S  T  S
                                    1  2  3  4  5  6  7  8  9
```

mbp23 tm3 stm3 mbp18

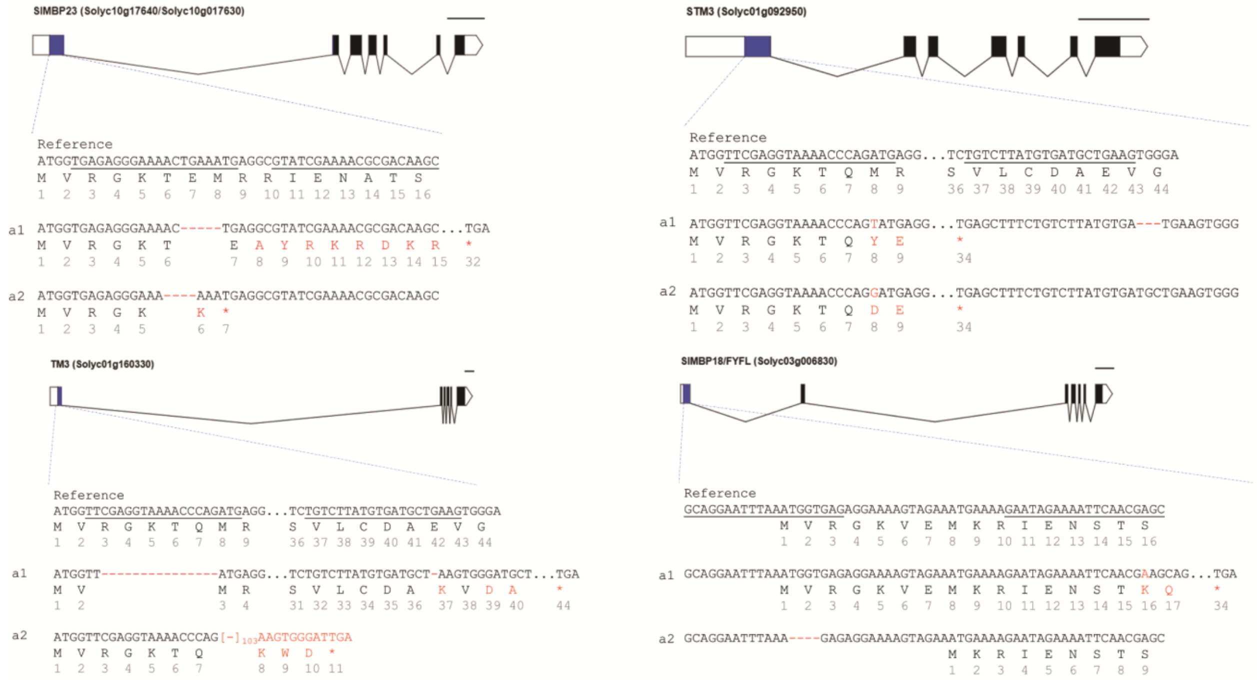

Supplementary Figure S3: Mutations of *SlSOC1* genes generated by CRISPR/Cas9 mutagenesis. The reference of the targeted region is shown with sgRNA targets underlined. The targeted exon of the gene is highlighted in blue, untargeted exons are black and the 5′ and 3′ UTRs are represented with white boxes. Insertions and deletions are marked in red. The scale bar above the intron/exon structure is 500 bp. The resulting translated protein sequence is shown under the nucleotide sequence. Numbers indicate amino acid number, with the start codon numbered as 1. Independent alleles are labeled by a1-a5. Identical alleles obtained from independent transformations are labeled as a1/a2. In case of multiallelic mutations in *STM3*, the four copies of the *stm3* allele are also numbered (e.g. a1.1-a1.4 is *stm3* mutant 1 with the different mutated alleles 1-4) and approximate allele frequency determined by pGEMt and/or pJET cloning is shown in blue next to the alleles.

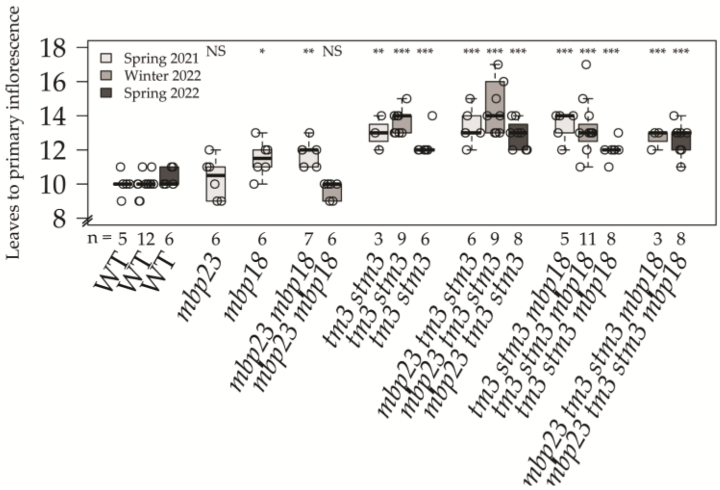

Supplementary Figure S4: Quantification of the primary transition to flowering in *slsoc1* mutants under greenhouse conditions. Shades of grey show three independent screenings. Some mutants were screened once, while others were screened in triplicate. Asterisks indicate statistical significant differences compared to its respective WT. NS, not significant.

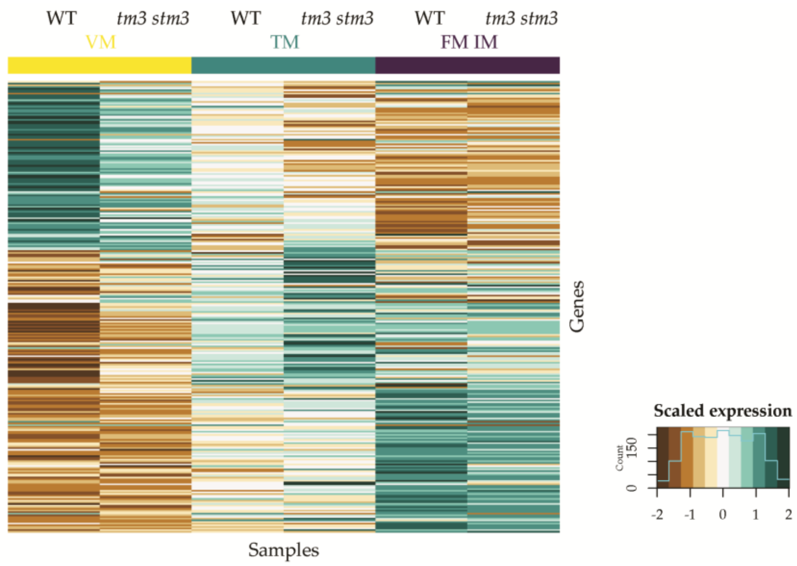

Supplementary Figure S5: Scaled expression of 288 markers genes for VM, TM and FM stages identified by Meir et al. [38] in WT and *tm3 stm3* RNA-Seq samples. Genes (rows) were ordered from early to late developmental stage from top to bottom. The Z-scaled expression represents the average FPKM value of three biological replicates. VM, vegetative meristem; TM, transition meristem; FM/IM, floral and inflorescence meristem.

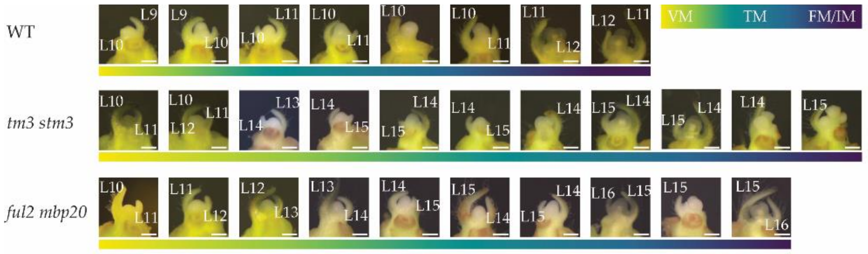

Supplementary Figure S6: Stereomicroscope images of reproductive meristem development in WT, *tm3 stm3* and *ful2 mbp20*. The color gradient under the images represents the gradual development of the meristems. The

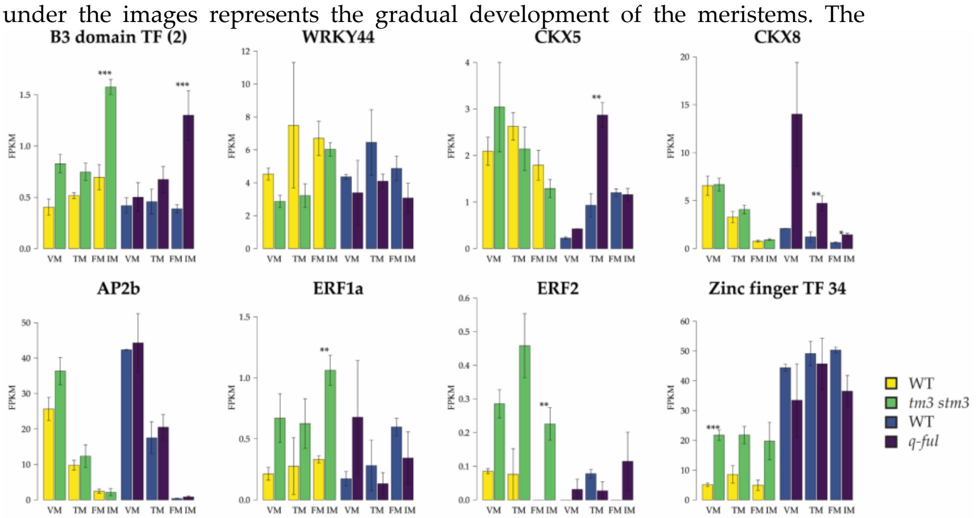

Supplementary Figure S7: Expression of several genes of interest in VM, TM and FM/IM of *tm3 stm3* and *q-ful*. Bars show mean FPKM values ± SEM. Asterisks indicate statistical significance according to the DESeq2 adjusted p values from the mutant compared to its respective WT. B3 domain TF (2), Solyc01g108930. FPKM, fragments per kilobase million; VM, vegetative meristem; TM, transition meristem; FM/IM, floral and inflorescence meristem.

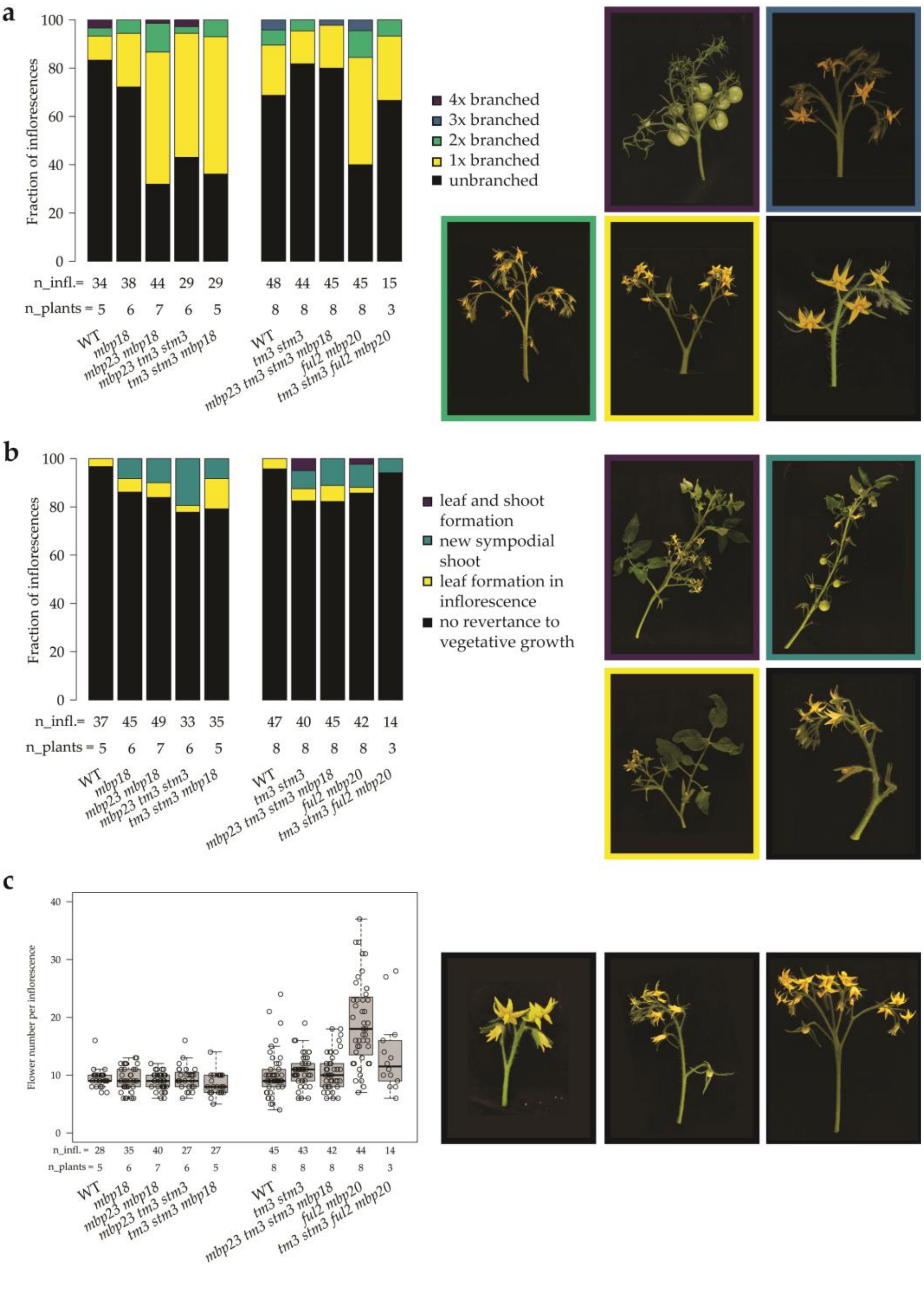

Supplementary Figure S8: Quantification of the inflorescence traits branching (**a**), revertance to vegetative growth (**b**), and flower number (**c**), in several *slsoc1* and *ful2 mbp20* mutants. The mutants were phenotyped in two independent screenings with separate WT controls. Representative inflorescences are shown next to the graphs. In the first screening in **b** (left 5 bars), no distinction was made between "leaf and sympodial shoot" and "new sympodial shoot", and all inflorescences of either category were scored as "new sympodial shoot".

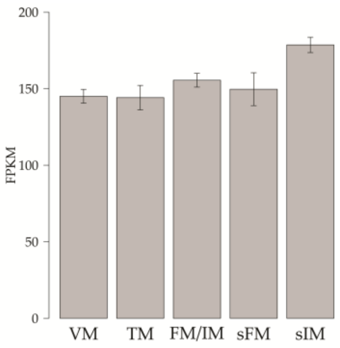

Supplementary Figure S9: Expression of *J* during reproductive meristem development. Bars show mean FPKM values of three biological replicates for VM, TM and FM/IM, and four biological replicates for sFM and sIM ± SEM. FPKM, fragments per kilobase million; VM, vegetative meristem; TM, transition meristem; FM/IM, floral and inflorescence meristem; sFM, sympodial floral meristem; sIM, sympodial inflorescence meristem.
